# The correlation between fine needle aspiration diagnosis and postoperative histopathological results of pediatric thyroid nodules based on the Bethesda system

**DOI:** 10.3389/fendo.2026.1750424

**Published:** 2026-06-01

**Authors:** Xue Bai, Jingjing Xu, Na Wei, Lin Xiao, Jinjuan Chen

**Affiliations:** Department of Pathology, First Affiliated Hospital of Zhengzhou University, Zhengzhou, Henan, China

**Keywords:** diagnosis, fine needle aspiration, pediatrics, risk of malignancy, thyroid nodules

## Abstract

**Background:**

Pediatric thyroid nodules have higher malignancy rates than adult nodules, necessitating accurate diagnostic evaluation. This study evaluated the correlation between fine needle aspiration (FNA) results using the Bethesda System for Reporting Thyroid Cytopathology (TBSRTC) and postoperative histopathological findings in pediatric patients.

**Methods:**

This retrospective analysis included 696 pediatric patients who underwent ultrasound-guided FNA for thyroid nodules from 2017-2024. Cytological results were classified according to TBSRTC 2023 criteria and correlated with histopathological outcomes in 246 surgically resected cases. Molecular testing included *BRAF* V600E mutation analysis on FNA specimens in non-operated patients, while *TERT* promoter mutations, *RAS* mutations, and *RET* fusions were assessed in formalin-fixed paraffin-embedded surgical specimens. Risk of malignancy (ROM) was calculated using conservative (ROM-C) and histological (ROM-H) approaches. Diagnostic performance was assessed for definitive cytological categories (II, IV, V, and VI).

**Results:**

Among 246 operated patients, the overall ROM ranged from 32.76% (ROM-C) to 92.68% (ROM-H). ROM increased progressively across Bethesda categories: II (1.11%-27.27%), III (25.40%-66.67%), IV (22.22%-100%), V (74.70%-100%), and VI (82.39%-100%). Papillary thyroid carcinoma predominated (88.62%). *BRAF* V600E mutation was detected in 51.53% of histologically confirmed malignancies and 10.96% in FNA samples from patients who did not undergo surgery. *RET* gene fusions were identified in 5.24% of malignant cases, *TERT* promoter mutations in 0.44%, while no *RAS* mutations were detected. Within definitive cytological categories (II, IV, V, and VI), the Bethesda system demonstrated sensitivity of 98.58%, specificity of 100%, and accuracy of 98.63%.

**Conclusion:**

The Bethesda system provides high diagnostic accuracy for definitive cytological categories in pediatric thyroid nodules. Even indeterminate categories carry elevated malignancy risk, supporting careful surgical consideration and the value of *BRAF* V600E testing for preoperative risk stratification.

## Introduction

Thyroid nodules are uncommon in children, with a prevalence of approximately 1–2% (1). Despite their rarity, the incidence of thyroid nodules developing into thyroid cancer in pediatric populations is higher compared to adults (2). Several factors are associated with an increased risk of thyroid cancer in pediatric patients, with prior head and neck radiotherapy being the most significant. Other factors include underlying thyroid disease, family history of thyroid cancer, genetic predisposition syndromes, and elevated serum TSH levels (3). Age and sex also influence the epidemiology and clinical behavior of pediatric thyroid cancer, with adolescent females demonstrating a higher incidence, whereas younger patients and males tend to present with more aggressive disease (4). Conversely, younger patients, particularly those aged 0–5 years, tend to have a lower incidence, with no malignant histology found in some studies within this age group. The most common presentation of pediatric thyroid cancer is asymptomatic thyroid nodules. Some cases of papillary thyroid cancer may also present with enlarged cervical lymph nodes, but these nodules are usually incidentally discovered during imaging studies or physical examinations (4, 5). In some cases, the diagnosis is made only after distant metastasis is identified. Given the higher malignancy rate and more advanced disease presentation in pediatric thyroid nodules, accurate evaluation of thyroid nodules is essential for guiding timely surgical intervention.

Fine needle aspiration (FNA) is a key diagnostic method used to evaluate thyroid nodules and differentiate between benign and malignant lesions (6). The Bethesda System for Reporting Thyroid Cytopathology (TBSRTC) is a widely accepted classification system that categorizes FNA results into six categories, ranging from non-diagnostic to malignant, providing a standardized approach for clinicians (7). While this system is well-established for adults, pediatric thyroid nodules exhibit different biological behavior. Studies suggested that pediatric patients with indeterminate FNA results, particularly those classified as Atypia of Undetermined Significance (AUS), may have a higher risk of malignancy compared to adults (8–10). For instance, a study by Sanchez et al. (8) found that 50% of AUS cases in pediatric patients were malignant upon surgical resection. These findings highlight the necessity of considering age when interpreting FNA results and planning follow-up actions.

Given the differences between pediatric and adult thyroid nodules, there is a need for further investigation into the correlation between FNA findings based on the Bethesda system and postoperative histopathological outcomes in children. This study aims to evaluate the correlation between FNA results using the Bethesda system and postoperative histopathological findings in pediatric thyroid nodules. Understanding this relationship is crucial for refining diagnostic and management strategies, ensuring appropriate treatment decisions, and minimizing unnecessary interventions in this unique patient population.

## Materials and methods

### Study design and subjects

This study was a retrospective analysis of pediatric patients who underwent ultrasound-guided FNA for thyroid nodules at the First Affiliated Hospital of Zhengzhou University from January 1, 2017, to December 31, 2024. Clinical data, FNA cytological results, and postoperative histopathological findings were retrieved from the hospital’s online medical records database. The study population was selected according to inclusion criteria encompassing: (1) aged≤ 18 years old; (2) underwent ultrasound-guided FNA for thyroid nodules; (3) complete clinical records.

### Indications for fine needle aspiration

FNA was performed according to the 2020 Chinese Guidelines for Ultrasound Malignancy Risk Stratification of Thyroid Nodules (C-TIRADS) (11). The indications for FNA included (1): C-TIRADS 3 nodules with a maximum diameter ≥2 cm (2); C-TIRADS 4A nodules with a maximum diameter ≥1.5 cm (3); C-TIRADS 4B–5 nodules with a maximum diameter ≥1 cm (4); nodules under regular surveillance demonstrating a >50% increase in solid component volume or at least two dimensions increasing by >20% (with maximum diameter >0.2 cm). For C-TIRADS 4B–5 nodules with a maximum diameter <1 cm, FNA was performed when any of the following conditions were present: prior to planned surgery or ablation therapy; multifocal suspicious nodules or nodules adjacent to the capsule, trachea, or recurrent laryngeal nerve; suspected cervical lymph node metastasis; abnormally elevated serum calcitonin levels; or a family history of thyroid cancer or thyroid cancer syndrome.

### Cytological classification and histopathological diagnosis

All FNA specimens underwent standardized processing utilizing ThinPrep liquid-based cytology technique followed by Papanicolaou staining protocols. Cytological interpretation was performed according to the 2023 Bethesda System for Reporting Thyroid Cytopathology, which categorizes results into six distinct diagnostic categories (7): TBSRTC I (Non-diagnostic), TBSRTC II (Benign), TBSRTC III (Atypia of Undetermined Significance), TBSRTC IV (Follicular Neoplasm), TBSRTC V (Suspicious for Malignancy), and TBSRTC VI (Malignant). For the purpose of this study, all FNA diagnoses were retrospectively reviewed and reclassified by two senior cytopathologists of associate professor level or above, employing double-blind methodology.

Postoperative histopathological examination served as the gold standard for diagnostic accuracy assessment throughout this investigation. Histopathological diagnoses were established through routine hematoxylin and eosin staining of formalin-fixed, paraffin-embedded tissue sections, with additional immunohistochemical studies performed when clinical or morphological features warranted further characterization. The histopathological evaluation process adhered to the 2022 World Health Organization Classification of Thyroid Neoplasms (12), ensuring accurate classification of benign and malignant lesions.

### Molecular genetic testing

DNA and RNA extraction from FNA samples and paraffin-embedded tissue specimens was performed according to manufacturer protocols (ADx-FFPE-DR50, Aide Biotechnology Co., Ltd. Xiamen, China). Extracted nucleic acids, negative controls, and positive controls were prepared in 5 μL aliquots and added to specifically configured PCR reaction tubes for subsequent analysis. Real-time PCR was performed on a SLAN-96S real-time fluorescence quantitative PCR system (Hongshi Medical Technology, Xiamen, China). *BRAF* V600E mutation detection and *TERT* gene promoter region mutations (C228T/C250T) were analyzed using Sanger sequencing combined with Arms-PCR methodology. Additional genetic alterations including *NRAS* gene exons 2/3/4 mutations, *HRAS* gene exon 3 mutations, *KRAS* gene exons 2/3/4 mutations, and *RET* gene fusions (CCDC6-RET/NCOA4-RET) were detected using fluorescence quantitative PCR with the Human *NRAS* Gene Mutation Detection Kit (National Standard: 20153401125). The detection sensitivity threshold was established at 1% mutant alleles within the total DNA content, with quality control measures ensuring reproducible results across all tested samples. *BRAF* V600E mutation analysis was specifically performed on cytological FNA specimens, while the remaining genetic analyses were conducted on histological paraffin-embedded tissue samples to optimize detection accuracy for each molecular target.

### Risk of malignancy calculation

Risk of malignancy (ROM) was calculated using two methodological approaches designed to provide ROM ranges for each TBSRTC category. For each TBSRTC category, the conservative ROM calculation (ROM-C) was determined using the formula: ROM-C = M/Total FNA cases within the category× 100%, where M represents malignant cases, providing a lower estimate of actual ROM. The histological ROM calculation (ROM-H) was determined using the formula: ROM-H = M/Total histological follow-up cases within the category × 100%, representing an upper estimate of actual ROM. Low-risk neoplasm (LRN), defined as tumors with uncertain malignant potential including follicular tumor of uncertain malignant potential (FT-UMP) and well-differentiated tumor of uncertain malignant potential (WDT-UMP), was specifically excluded from malignant tumor classification for ROM calculations, following current consensus guidelines (12). The actual ROM was considered to fall within the range established by ROM-C and ROM-H values.

### Statistical analysis

All performance metrics were calculated with 95% confidence intervals to assess diagnostic accuracy. Statistical analysis was performed using SPSS version 26.0 (IBM Corp., Armonk, NY, USA), with P-values less than 0.05 considered statistically significant for all comparative analyses. Normality of continuous variables was assessed using the Kolmogorov-Smirnov test. Continuous variables were presented as means ± standard deviations for normally distributed data or medians with interquartile ranges for non-normally distributed data. Categorical variables were expressed as frequencies and percentages (%). Categorical variables were analyzed using Pearson’s chi-square test or Fisher’s exact test when expected cell counts were less than five.

True positive (TP), false positive (FP), true negative (TN), and false negative (FN) cases were defined according to the following criteria: TP cases encompassed histologically malignant nodules with cytological classification of IV, V, or VI. FN cases included histologically malignant nodules with cytological classification of II. TN cases comprised histologically benign nodules with cytological classification of II, while FP cases encompassed histologically benign nodules with cytological classification of IV, V, or VI. Sensitivity, specificity, positive predictive value (PPV), and negative predictive value (NPV) were calculated.

## Results

### Study cohort

A total of 696 patients who underwent FNA were included in this study, comprising 501 females and 195 males, with age ranging from 3 to 18 years (mean age: 14.35 ± 3.28 years). In these patients, 824 FNA procedures were performed, and 113 patients underwent two or more repeat biopsies. The main reasons for repeated procedures included: nondiagnostic samples at the initial exam (Bethesda category I), cytological findings of “atypia of undetermined significance” or “follicular lesion of undetermined significance” (Bethesda category III), and the persistence or emergence of highly suspicious ultrasound features. Their detailed characteristics including initial and final TBSRTC categories, number of FNA procedures, and surgical outcomes are presented in **Supplementary Table 1**. For patients who underwent repeated examinations, the TBSRTC category was recorded according to the highest grade. Among the 696 patients, 246 (35.44%) underwent surgery with histopathological examination, and 422 (60.63%) received molecular genetic testing. **Table 1** summarizes the distribution of TBSRTC categories based on the Bethesda System.

**Table 1:** Distribution of TBSRTC categories.

TBSRTC categories	Age (IQR)	Sex (F/M)	Cytological results, n (%)	Histological follow-up, n (%)	Histological follow-up/FNA (%)
I	14 (4.0)	66/28	94 (13.51%)	2 (0.82%)	2.13%
II	14 (5.0)	193/78	271 (38.94%)	11 (4.51%)	4.06%
III	15 (4.5)	51/12	63 (9.05%)	24 (9.84%)	38.10%
IV	12 (6.0)	9/0	9 (1.29%)	2 (0.82%)	22.22%
V	15.5 (5.0)	64/19	83 (11.93%)	62 (25.20)	74.70%
VI	16 (4.0)	118/58	176 (25.29)	145 (59.43)	82.39%
Total	15 (4.0)	501/195	696	246	35.06%

### Histopathological Results and ROM

**Table 2** demonstrates the correlation between TBSRTC cytological categories and histopathological outcomes in 246 pediatric patients who underwent surgical resection. The ROM showed a progressive increase across TBSRTC categories. TBSRTC I cases showed 1 malignant case and 1 LRN among the 2 cases with histological follow-up (ROM-C: 1.06%, ROM-H: 50.00%). TBSRTC II demonstrated a low malignancy rate with 3 malignant cases out of 11 cases (ROM-C: 1.11%, ROM-H: 27.27%). TBSRTC III revealed a substantially elevated malignancy risk, with 16 malignant cases among 24 patients (ROM-C: 25.40%, ROM-H: 66.67%). TBSRTC IV showed 2 malignant cases out of 2 cases with histological follow-up (ROM-C: 22.22%, ROM-H: 100%). TBSRTC V and TBSRTC VI categories demonstrated consistently high malignancy rates, with ROM-C values of 74.70% and 82.39%, respectively, and ROM-H values of 100% for both categories.

**Table 2:** Histopathological outcomes and risk of malignancy for pediatric thyroid nodules according to TBSRTC categories.

TBSRTC categories	Histological follow-up results, n(%) (n=246)			ROM (%)	
	M	LRN	B	ROM-C	ROM-H
I	1 (0.41%)	1 (0.41%)	–	1.06%	50.00%
II	3 (1.22%)	1 (0.41%)	7 (2.85%)	1.11%	27.27%
III	16 (6.50%)	2 (0.81%)	6 (2.44%)	25.40%	66.67%
IV	2 (0.81%)	–	–	22.22%	100.00%
V	62 (25.20%)	–	–	74.70%	100.00%
VI	145 (58.94%)	–	–	82.39%	100.00%
Total	229 (93.09%)	4 (1.63%)	13 (5.28%)	32.76%	92.68%

M, Malignant; LRN, Low-risk neoplasm; B, Benign; ROM-C, Conservative risk of malignancy; ROM-H, Histological risk of malignancy. Dash (–) indicates no cases in that category.

LRN were identified in 4 cases, predominantly in TBSRTC I (n=1) and TBSRTC II (n=1) and TBSRTC III (n=2) categories. The overall malignancy rate was 32.76% (ROM-C) when calculated from the total FNA cohort and 92.68% (ROM-H) when calculated from cases with histological follow-up. The two ROM calculation methodology provides a range within which the actual malignancy risk likely resides, with ROM-C representing a conservative underestimate and ROM-H representing an upper estimate due to selective surgical intervention in higher-risk cases.

### Surgical procedures

The distribution of surgical procedures across TBSRTC categories is presented in **Supplementary Table 2**. Among the 246 patients who underwent surgery, total thyroidectomy was performed in 173 patients (70.33%), while lobectomy was performed in 73 patients (29.67%). The rate of total thyroidectomy was highest in TBSRTC categories V (80.65%) and VI (73.10%). In TBSRTC category II, lobectomy was more frequently performed (72.73%). For indeterminate categories III and IV, total thyroidectomy and lobectomy were evenly distributed (all 50.00%).

### Histopathological subtypes and molecular characteristics

**Table 3** demonstrates the distribution of histopathological subtypes across different TBSRTC categories among 246 pediatric patients who underwent surgical resection. Papillary thyroid carcinoma (PTC) was the predominant malignant subtype, accounting for 218 cases (88.62%), with the majority concentrated in TBSRTC categories V (23.58%) and VI (58.54%). Follicular thyroid carcinoma (FTC) was identified in 4 cases (1.63%), distributed across TBSRTC categories II, III, and IV. Medullary thyroid carcinoma (MTC) represented 3 cases (1.22%), occurring exclusively in higher-risk categories (V and VI). Benign lesions comprised 17 cases (6.91%), including 4.88% of follicular nodular disease (FND) and 0.41% of follicular adenoma (FA). Follicular tumors of uncertain malignant potential (FT-UMP) were identified in 4 cases (1.63%). LRN accounted for 4 cases (1.63%), predominantly found in lower TBSRTC categories (I, II, and III). The “Others” category included 4 rare cases (1.63%): 3 cases of poorly differentiated/high-grade thyroid carcinoma identified in TBSRTC categories II, III, and V, and 1 case of Langerhans cell histiocytosis in TBSRTC category V. Notably, malignant lesions were identified across all TBSRTC categories, including 3 cases in the presumably benign category II. It also worth mentioning that all 3 MTC cases were admitted due to presence of palpable neck mass. Serum calcitonin was elevated in 2 patients, while one did not undergo preoperative testing. One patient had a family history of MTC. None underwent RET germline mutation screening before surgeries.

**Table 3:** Distribution of histopathological subtypes across TBSRTC categories.

TBSRTC categories	B		LRN	M			
	FND	FA	FT-UMP	PTC	FTC	MTC	Others
I	–	–	1 (0.41%)	1 (0.41%)	–	–	–
II	7 (2.85%)	–	1 (0.41%)	1 (0.41%)	1 (0.41%)	–	1 (0.41%)
III	5 (2.03%)	1 (0.41%)	2 (0.81%)	13 (5.28%)	2 (0.81%)	–	1 (0.41%)
IV	–	–	–	1 (0.41%)	1 (0.41%)	–	–
V	–	–	–	58 (23.58%)	–	2 (0.81%)	2 (0.81%)
VI	–	–	–	144 (58.54%)	–	1 (0.41%)	–
Total	12 (4.88%)	1 (0.41%)	4 (1.63%)	218 (88.62%)	4 (1.63%)	3 (1.22%)	4 (1.63%)

B, Benign; LRN, Low-risk neoplasm; M, Malignant; FND, Follicular nodular disease/hyperplastic nodule; FA, Follicular adenoma; FT-UMP, Follicular tumor of uncertain malignant potential; PTC, Papillary thyroid carcinoma; FTC, Follicular thyroid carcinoma; MTC, Medullary thyroid carcinoma. Dash (–) indicates no cases in that category.

In addition, **Table 4** presents the distribution of mutations detected in thyroid nodules, stratified by sample type and histopathological outcome. Among 450 patients who did not undergo surgery, *BRAF* V600E mutation analysis was performed on FNA cytological specimens from 219 patients, revealing a positivity rate of 10.96% (24/219 cases). For the 246 patients with surgical resection, molecular testing was conducted on histological tissue samples, including *TERT* promoter mutations, *RAS* mutations (*NRAS, HRAS, KRAS*), and *RET* gene fusions. *BRAF* V600E mutation was the most prevalent molecular alteration, detected in 118 of 229 malignant cases (51.53%), while absent in all LRN (0/4) and benign lesions (0/13). *TERT* promoter mutations were rare, identified in only 1 malignant case (0.44%). Interestingly, no *RAS* mutations (including *KRAS, HRAS*, and *NRAS*) were detected in any of the histologically examined cases. *RET* gene fusions were found exclusively in malignant lesions, accounting for 12 of 229 cases (5.24%). None of the molecular alterations tested were identified in LRN or benign lesions, supporting the diagnostic utility of these markers in distinguishing malignant from non-malignant thyroid nodules in pediatric patients.

**Table 4:** Distribution of mutations in thyroid nodules.

Categories	Total, n	*BRAF* V600E, n (%)	*TERT* promoter, n (%)	*RAS* mutations, n (%)	*RET* fusions, n (%)
FNA^a^	219	24 (10.96%)	/	/	/
Histopathological					
M	229	118 (51.53%)	1 (0.44%)	–	12 (5.24%)
LRN	4	–	–	–	–
B	13	–	–	–	–

B, Benign; LRN, Low-risk neoplasm; M, Malignant; FNA, Fine needle aspiration; BRAF, B-Raf proto-oncogene, serine/threonine kinase; TERT, Telomerase reverse transcriptase; RAS, Rat sarcoma viral oncogene homolog; RET, Rearranged during transfection proto-oncogene; Slash (/) indicates testing not performed on FNA specimens. Dash (–) indicates no cases in that category.

^a^ *BRAF* V600E mutation analysis was performed on FNA cytological specimens from patients who did not undergo surgical resection.

### Diagnostic performance of TBSRTC categories

**Table 5** presents the diagnostic performance of FNA using the TBSRTC system for differentiating malignant from non-malignant thyroid nodules in pediatric patients. The analysis was based on 219 cases with definitive histological outcomes, excluding TBSRTC categories I and III to provide a binary classification assessment. FNA demonstrated excellent diagnostic performance with a sensitivity of 98.58% (95% CI: 96.99-100.00%) and perfect specificity of 100.00% (95% CI: 59.04-100.00%). The positive predictive value was 100.00% (95% CI: 98.25-100.00%), indicating that all cases classified as malignant by FNA (TBSRTC IV, V, VI) were confirmed as malignant on histological examination. The negative predictive value was 70.00% (95% CI: 41.60-98.40%), with 3 false negative cases occurring in the TBSRTC II category. The overall diagnostic accuracy reached 98.63% (95% CI: 97.08-100.00%), demonstrating the high reliability of the TBSRTC system in pediatric thyroid nodule evaluation when applied to definitive diagnostic categories.

**Table 5:** Diagnostic performance of FNA using TBSRTC.

FNA result	Histological outcome		Performance metrics	Value (%)	95% CI
	Non-malignant	Malignant			
Non-malignant	7	3	Sensitivity	98.58	96.99-100.00
Malignant	0	209	Specificity	100	59.04-100.00
Total	7	212	PPV	100	98.25-100.00
			NPV	70	41.60-98.40
			Accuracy	98.63	97.08-100.00

CI, Confidence Interval; PPV, Positive Predictive Value; NPV, Negative Predictive Value.

## Discussion

This study analyzed the correlation between TBSRTC cytological categories and histopathological outcomes in pediatric thyroid nodules. The key findings demonstrate that pediatric thyroid nodules exhibit substantially higher malignancy rates across all TBSRTC categories compared to adult populations, with particularly notable differences in the indeterminate categories. The overall ROM ranged from 32.76% to 92.68%, with TBSRTC category III showing a remarkably high ROM of 25.40%-66.67%. PTC was the predominant malignancy, accounting for 88.62% of cases, and *BRAF* V600E mutation was detected in over half of histologically confirmed malignancies. The TBSRTC system demonstrated excellent diagnostic performance within definitive cytological categories (II, IV, V, and VI).

The elevated ROM observed in indeterminate categories aligned with previous pediatric studies showing higher malignancy rates compared to adults with equivalent cytological findings (13). A large multicenter literature review reported ROM values for Bethesda categories: 7.2% for category II, 29.6% for category III, 42.3% for category IV, 90.8% for category V, and 98.8% for category VI in pediatric populations, demonstrating consistency with our findings (14). The TBSRTC category III ROM in our histologically confirmed pediatric cohort was 66.7%, which is markedly higher than the 10–30% from adult populations (15), and consistent with recent pediatric series that have documented ROMs of 50–67% (16–18). However, the interpretation of ROM values requires consideration of the surgical resection rates across different categories. For categories V and VI, ROM-H is more likely to represent the true malignancy risk given the high surgical rates (74.70% and 82.39%, respectively). In contrast, for category III, where only 38.10% of patients underwent surgery, the actual ROM likely falls between ROM-C (25.40%) and ROM-H (66.67%), as surgical decisions were influenced by additional clinical and sonographic concerns that enriched for higher-risk cases. Category IV had very few cases with only 2 patients undergoing surgery, precluding meaningful conclusions about ROM in this category. These differences have critical implications for management strategies, as adult guidelines often recommend repeat FNA or molecular testing for category III nodules before surgical intervention, while pediatric patients may benefit from more definitive approaches. The biological basis for these differences remains incompletely understood but may relate to distinct molecular landscapes between pediatric and adult thyroid cancers.

Several factors may contribute to these differences. The pediatric thyroid gland is in an active phase of growth and differentiation, rendering thyrocytes more susceptible to oncogenic transformation from environmental stimuli such as ionizing radiation and activating mutations (2, 19). Pediatric thyroid cancer differs substantially from that of adults, with a higher prevalence of fusion oncogenes (*RET/PTC*, *NTRK*) that are associated with more advanced disease at presentation and higher rates of persistent or recurrent disease (20, 21). *BRAF* V600E-driven MAPK pathway activation, detected in 51.53% of malignancies in this cohort, may exert more potent oncogenic effects in the developing thyroid microenvironment, given the heightened proliferative state of pediatric thyrocytes (22). Additionally, clinical selection bias likely contributes: pediatric patients typically undergo FNA only after repeated ultrasound surveillance confirming suspicious features, thereby enriching the evaluated cohort for higher-risk nodules (2).

The high ROM observed in TBSRTC category III nodules in this pediatric cohort (25.40%–66.67%) may provide important implications for clinical management. Current adult-based guidelines recommend repeat FNA or active surveillance for category III nodules, based on a reported ROM of 10–30% (15). However, the findings in this study, consistent with other recent pediatric series, suggest that these conservative strategies may be insufficient for pediatric patients (23, 24). The substantially elevated ROM supports a lower threshold for surgical intervention or, alternatively, the routine integration of molecular testing for risk stratification in pediatric category III nodules. *BRAF* V600E testing may serve as a practical triage tool, with positive results prompting direct surgical referral (25). The 2022 European Thyroid Association guidelines have acknowledged the need for age-specific management considerations, and data from this study further support the development of pediatric-specific algorithms for indeterminate thyroid cytology that incorporate both clinical risk factors and molecular parameters (5).

The surgical rate was relatively low in Bethesda II category (4.06%, 11/271), as surgery was reserved for patients with compressive symptoms due to large nodule size, progressive nodule enlargement during follow-up, development of suspicious ultrasonographic features, or concurrent thyroid disease requiring surgical management. Notably, all 3 malignant cases in this category occurred in patients who had either compressive symptoms or highly suspicious ultrasound findings with suspected lymph node involvement, supporting the importance of integrating clinical and sonographic findings with cytological results. The relatively high ROM-H (27.27%) in category II therefore reflects selection bias rather than true malignancy risk of benign cytology.

It should be noted that the diagnostic performance analysis was restricted to 219 cases with definitive histological outcomes (TBSRTC categories II, IV, V, and VI), excluding categories I and III. The surgery-enriched nature of the cohort structurally reduces the likelihood of false-positive results, as benign nodules classified as categories IV–VI are rarely encountered in surgical series. The diagnostic performance metrics in our study, with sensitivity of 98.58% and specificity of 100%, are comparable to or exceed those reported in earlier pediatric surgical series, where sensitivities ranged from 90% to 96% and specificities from 75% to 96%, yielding an overall accuracy of approximately 90% (26, 27). Similar to our findings, another recent pediatric cohort reported accuracy of 99% with sensitivity of 94% and specificity of 100%, suggesting that FNA can achieve excellent diagnostic performance in pediatric populations when applied to definitive diagnostic categories (28). The superior diagnostic performance in pediatric cohorts may reflect the well-differentiated nature of most pediatric thyroid malignancies, which typically present with classic cytological features of papillary thyroid carcinoma including nuclear grooves, chromatin clearing, and intranuclear pseudoinclusions that are readily identifiable on cytology. The high negative predictive value in pediatric studies, ranging from 98% to 100%, provides reassurance that benign FNA diagnoses reliably exclude malignancy, though the three false-negative cases in our TBSRTC II category underscore the importance of clinical and sonographic correlation even with benign cytology (28). These performance characteristics support the continued use of TBSRTC-based FNA as the primary diagnostic modality for pediatric thyroid nodules while acknowledging the need for heightened vigilance in indeterminate categories and integration with clinical, sonographic, and molecular data for optimal risk stratification.

The molecular findings provided important insights into pediatric thyroid cancer pathogenesis. A large pediatric cohort study reported *BRAF* V600E mutation rates of 57.4% in PTC, with higher incidence in the 13–18 age group compared to 6–12 years (29). Our detection rate of 51.53% falls within this range and is notably lower than the approximately 70% observed in adult PTC (30, 31). Research comparing pediatric and adult populations found *BRAF* V600E mutations occur in children at rates comparable to adults, though without clear evidence that the mutation correlates with more extensive disease in pediatric cases (32). The significantly higher BRAF V600E positivity rate in histologically confirmed malignancies (51.53%) compared to FNA samples from unoperated patients (10.96%) reflects the enrichment of malignant lesions among surgically resected cases. Notably, among 24 patients with BRAF V600E mutation detected on FNA who did not undergo thyroidectomy, 3 underwent radiofrequency ablation, 2 declined surgical intervention, 1 was unsuitable for surgery due to severe comorbidity, and the remaining 18 were lost to follow-up. The intermediate prevalence in our cohort likely reflects the unique molecular landscape of pediatric thyroid cancer, where fusion oncogenes play a more prominent role than in adults. A genomic analysis of 131 pediatric thyroid cancers identified *RET/NTRK* fusions in 32.8% of tumor samples, with these fusions more common in patients younger than 10 years (91%) compared to older pediatric patients (27%) (20). Our detection of *RET* fusions in 5.24% of malignant cases, while lower than some reported series, reflects the technical challenges in fusion detection and possible cohort differences. RET fusion genes were detected in 11.4% of PTC in a large international cohort, occurring exclusively in PTC and not in benign thyroid tissue, supporting our finding that these alterations are specific for malignancy (33). Importantly, fusion oncogene-positive PTCs in pediatric patients were associated with younger age, more advanced disease stage, and higher rates of recurrent or persistent disease compared to BRAF-mutant tumors, highlighting the prognostic significance of molecular characterization (34). The absence of RAS mutations in our cohort contrasts with adult data where these mutations account for 10-20% of thyroid malignancies, further emphasizing the distinct molecular profile of pediatric disease and suggesting different pathways of tumorigenesis.

No cases in our cohort met the diagnostic criteria for NIFTP according to the 2022 WHO Classification. This finding is consistent with the known low prevalence of NIFTP in Asian populations (0–5%) compared to Western series (10–20%), attributed to different diagnostic thresholds for PTC-type nuclear features and distinct clinical management approaches (35). NIFTP appears to be even rarer in pediatric populations, with a recent study reporting only 1.1% (3/262) among surgically resected pediatric thyroid nodules (36). The molecular profile of our cohort, characterized by high *BRAF* V600E positivity and absence of *RAS* mutations, further supports the predominance of *BRAF*-driven classic PTC rather than *RAS*-lineage follicular-patterned tumors from which NIFTP typically arises (37).

This study contributes to the existing pediatric thyroid literature in several ways. It represents a large single-center pediatric thyroid FNA cohort evaluated using the updated 2023 TBSRTC criteria, providing contemporary ROM data across all Bethesda categories. Additionally, it offers cytological and molecular characterization from an East Asian pediatric population, which remains underrepresented in the published literature. The integration of TBSRTC cytological classification with multiple molecular markers (*BRAF* V600E, *TERT*, *RAS*, *RET*) within a single cohort allows for a more comprehensive diagnostic assessment compared to studies reporting cytological or molecular data alone. Based on these findings, we suggest a tiered clinical approach: for definitive categories (II, IV, V, VI), the Bethesda system provides reliable risk stratification; for indeterminate categories, particularly category III, molecular testing may be considered to inform surgical decision-making in pediatric patients.

This study has several limitations that warrant consideration. The retrospective design may introduce selection bias, as surgical resection was not uniformly performed across all FNA cases, with the operated cohort likely representing higher-risk patients and potentially inflating ROM-H calculations. Furthermore, the surgery-enriched cohort structurally inflates specificity, as benign nodules classified as categories IV–VI are rarely resected in clinical practice; therefore, the reported specificity of 100% and accuracy of 98.63% should be interpreted as applicable only to definitive diagnostic categories and not extrapolated to the full clinical spectrum. The relatively high proportion of nondiagnostic FNA results (94 cases, 13.51%) with limited repeat aspiration and surgical follow-up data restricts our ability to determine outcomes in this category. The very small sample size of TBSRTC category IV (n=9) with only 2 surgical cases precludes meaningful conclusions about ROM. Most importantly, the surgical resection rate for category III was less than half (38.10%), which likely inflates ROM-H values due to preferential surgery in patients with additional concerning features. Therefore, the ROM values reported, particularly ROM-H for indeterminate categories, should be interpreted with caution as they may overestimate the true malignancy risk. Molecular testing was not performed on all specimens, limiting characterization of the molecular landscape. As a single-center study from a tertiary referral hospital, the findings may not be generalizable to all pediatric populations or community practice settings. Long-term follow-up data were not available to assess the prognostic implications of different TBSRTC categories or molecular alterations. Notably, among 24 *BRAF* V600E-positive patients who did not undergo surgery, 18 were lost to follow-up, precluding definitive conclusions regarding the clinical utility of molecular markers in guiding non-surgical management. Future prospective multicenter studies with standardized surgical criteria, comprehensive molecular profiling on all cases, and extended follow-up, and standardized tracking of molecular-positive non-operated patients would provide more definitive data. The development of pediatric-specific modifications to the TBSRTC system, particularly for category III lesions given their elevated malignancy risk, could improve diagnostic accuracy and clinical decision-making in this unique population.

## Conclusion

Pediatric thyroid nodules exhibit elevated malignancy rates across all categories compared to adults, with TBSRTC III showing ROM of 25.40%–66.67%. Within definitive cytological categories (II, IV, V, and VI), the Bethesda system demonstrates high diagnostic accuracy, though the surgery-enriched cohort may limit generalizability. These findings support more aggressive surgical management for pediatric indeterminate nodules and highlight the value of BRAF V600E testing for preoperative risk stratification. However, substantial loss to follow-up among BRAF V600E-positive non-operated patients limits conclusions regarding molecular testing in non-surgical management. Prospective multicenter studies with standardized follow-up protocols are warranted.

## Data Availability

The original contributions presented in the study are included in the article/**Supplementary Material**. Further inquiries can be directed to the corresponding author.

## References

[1] Richman DM Benson CB Doubilet PM Peters HE Huang SA Asch E . Thyroid nodules in pediatric patients: Sonographic characteristics and likelihood of cancer. Radiology. (2018) 288:591–9. doi: 10.1530/ey.15.3.12. PMID: 29714678 PMC6369930

[2] Lai ST Bauer AJ . Approach to the pediatric patient with thyroid nodules. J Clin Endocrinol Metab. (2025) 110:2339–52. doi: 10.1210/clinem/dgaf090. PMID: 39943817 PMC12261091

[3] Francis GL Waguespack SG Bauer AJ Angelos P Benvenga S Cerutti JM . Management guidelines for children with thyroid nodules and differentiated thyroid cancer. Thyroid: Off J Am Thyroid Assoc. (2015) 25:716–59. doi: 10.1089/thy.2014.0460. PMID: 25900731 PMC4854274

[4] Sandy JL Titmuss A Hameed S Cho YH Sandler G Benitez-Aguirre P . Thyroid nodules in children and adolescents: Investigation and management. J Paediatrics Child Health. (2022) 58:2163–8. doi: 10.1111/jpc.16257. PMID: 36382588 PMC10099987

[5] Lebbink CA Links TP Czarniecka A Dias RP Elisei R Izatt L . 2022 European Thyroid Association guidelines for the management of pediatric thyroid nodules and differentiated thyroid carcinoma. Eur Thyroid J. (2022) 11:e220146. doi: 10.1530/ey.20.1.13. PMID: 36228315 PMC9716393

[6] Feldkamp J Führer D Luster M Musholt TJ Spitzweg C Schott M . Fine needle aspiration in the investigation of thyroid nodules. Deutsches Arzteblatt Int. (2016) 113:353–9. doi: 10.3238/arztebl.2016.0353. PMID: 27294815 PMC4906830

[7] Ali SZ Baloch ZW Cochand-Priollet B Schmitt FC Vielh P VanderLaan PA . The 2023 bethesda system for reporting thyroid cytopathology. Thyroid: Off J Am Thyroid Assoc. (2023) 33:1039–44. doi: 10.1016/j.jasc.2023.05.005. PMID: 37427847

[8] Ixchel Sanchez S Abbasi S Robinson MT Maleki Z . Pediatric thyroid nodules: A comprehensive study of cytology, histology, molecular findings, and risk of Malignancy with emphasis on atypia of undetermined significance category. Am J Clin Pathol. (2025) 164:207–15. doi: 10.1093/ajcp/aqaf034. PMID: 40328656

[9] Jin X Jing X Smola B Heider A . Malignant risk of pediatric Bethesda category III thyroid nodules subcategorized by nuclear atypia and other: A single institution experience. Cancer Cytopathol. (2024) 132:564–8. doi: 10.1002/cncy.22831. PMID: 38771850

[10] Elmaoğulları S Özalkak Ş Çetinkaya S Karaman İ Üner Ç Arda N . Evaluation of children and adolescents with thyroid nodules: A single center experience. J Clin Res Pediatr Endocrinol. (2021) 13:276–84. doi: 10.4274/jcrpe.galenos.2020.2020.0213 PMC8388051 33374093

[11] Zhou J Yin L Wei X Zhang S Song Y Luo B . 2020 Chinese guidelines for ultrasound Malignancy risk stratification of thyroid nodules: The C-TIRADS. Endocrine. (2020) 70:256–79. doi: 10.1007/s12020-020-02441-y. PMID: 32827126

[12] Baloch ZW Asa SL Barletta JA Ghossein RA Juhlin CC Jung CK . Overview of the 2022 WHO classification of thyroid neoplasms. Endocr Pathol. (2022) 33:27–63. doi: 10.1007/s12022-022-09707-3. PMID: 35288841

[13] Cherella CE Angell TE Richman DM Frates MC Benson CB Moore FD . Differences in thyroid nodule cytology and Malignancy risk between children and adults. Thyroid: Off J Am Thyroid Assoc. (2019) 29:1097–104. doi: 10.1089/thy.2018.0728. PMID: 31298618 PMC6707031

[14] Hess JR Van Tassel DC Runyan CE Morrison Z Walsh AM Schafernak KT . Performance of ACR TI-RADS and the Bethesda System in predicting risk of Malignancy in thyroid nodules at a large children’s hospital and a comprehensive review of the pediatric literature. Cancers. (2023) 15:3975. doi: 10.3390/cancers15153975. PMID: 37568791 PMC10417028

[15] Ho AS Sarti EE Jain KS Wang H Nixon IJ Shaha AR . Malignancy rate in thyroid nodules classified as Bethesda category III (AUS/FLUS). Thyroid: Off J Am Thyroid Assoc. (2014) 24:832–9. doi: 10.1089/thy.2013.0317. PMID: 24341462 PMC4948206

[16] Tuli G Munarin J Biga A Quaglino F Carbonaro G De Sanctis L . Toward standardized management of indeterminate thyroid nodules in pediatric patients: A systematic review and call for a comprehensive risk stratification model. J Clin Med. (2025) 14:6112. doi: 10.3390/jcm14176112. PMID: 40943872 PMC12429790

[17] Vuong HG Chung DGB Ngo LM Bui TQ Hassell L Jung CK . The use of the Bethesda System for Reporting Thyroid Cytopathology in pediatric thyroid nodules: A meta-analysis. Thyroid: Off J Am Thyroid Assoc. (2021) 31:1203–11. doi: 10.1089/thy.2020.0702. PMID: 33504264

[18] Canberk S Barroca H Girão I Aydın O Uguz A Erdogan K . Performance of the Bethesda System for Reporting Thyroid Cytology in multi-institutional large cohort of pediatric thyroid nodules: A detailed analysis. Diagnostics (Basel Switzerland). (2022) 12:3975. doi: 10.3390/diagnostics12010179. PMID: 35054346 PMC8774335

[19] Gallant JN Chen SC Ortega CA Rohde SL Belcher RH Netterville JL . Evaluation of the molecular landscape of pediatric thyroid nodules and use of a multigene genomic classifier in children. JAMA Oncol. (2022) 8:1323–7. doi: 10.1530/ey.20.1.5. PMID: 35679040 PMC9185516

[20] Franco AT Ricarte-Filho JC Isaza A Jones Z Jain N Mostoufi-Moab S . Fusion oncogenes are associated with increased metastatic capacity and persistent disease in pediatric thyroid cancers. J Clin Oncol. (2022) 40:1081–90. doi: 10.1530/ey.19.3.14. PMID: 35015563 PMC8966969

[21] Satapathy S Bal C . Genomic landscape of sporadic pediatric differentiated thyroid cancers: A systematic review and meta-analysis. J Pediatr Endocrinol Metabolism: JPEM. (2022) 35:749–60. doi: 10.1515/jpem-2021-0741. PMID: 35434981

[22] Pintos SLA Varela F Jaén A Guillermo A Lobos PA Liberto DH . The Bethesda System for Reporting Thyroid Cytopathology: Risk of Malignancy in pediatric thyroid nodules. J Pediatr Surg. (2025) 60:162126. doi: 10.1016/j.jpedsurg.2024.162126. PMID: 39793534

[23] Aekka A Zimmerman D Josefson JL . An updated review on the use of fine needle aspiration biopsy in pediatric thyroid disease. Endocrine Practice: Off J Am Coll Endocrinol Am Assoc Clin Endocrinologists. (2025) 31:1346–9. doi: 10.1016/j.eprac.2025.08.005. PMID: 40816523

[24] Aekka A Arva NC Ren HZ Raval M Rastatter J Finkelstein S . Multiplatform molecular testing as an adjunct to fine needle aspiration in the evaluation of pediatric thyroid nodules. J Clin Endocrinol Metab. (2025) 110:e3738–44. doi: 10.1210/clinem/dgaf110. PMID: 40037628

[25] Vanderniet JA Fuentes-Bolanos NA Cho YH Chung DKV Sandler G Moghimi A . Recent advances in diagnostics and therapeutics for paediatric thyroid cancer. J Paediatrics Child Health. (2025) 61:666–75. doi: 10.1111/jpc.70013. PMID: 39934993 PMC12053232

[26] Stevens C Lee JKP Sadatsafavi M Blair GK . Pediatric thyroid fine-needle aspiration cytology: A meta-analysis. J Pediatr Surg. (2009) 44:2184–91. doi: 10.1016/j.jpedsurg.2009.07.022. PMID: 19944231

[27] Mirshemirani A Roshanzamir F Tabari AK Ghorobi J Salehpoor S Gorji FA . Thyroid nodules in childhood: A single institute experience. Iranian J Pediatr. (2010) 20:91–6. PMC3446001 23056688

[28] Partyka KL Huang EC Cramer HM Chen S Wu HH . Histologic and clinical follow-up of thyroid fine-needle aspirates in pediatric patients. Cancer Cytopathol. (2016) 124:467–71. doi: 10.1016/j.jasc.2015.09.189. PMID: 26970342

[29] Li Y Wang Y Li L Qiu X . The clinical significance of BRAFV600E mutations in pediatric papillary thyroid carcinomas. Sci Rep. (2022) 12:12674. doi: 10.1038/s41598-022-16207-1. PMID: 35879379 PMC9314322

[30] Wang Q Zhao N Zhang J . Gene mutation analysis in papillary thyroid carcinoma using a multi-gene panel in China. Int J Gen Med. (2021) 14:5139–48. doi: 10.2147/ijgm.s327409. PMID: 34511996 PMC8421255

[31] Brumfield A Azar SA Nordgren R Cohen RN Sarne D Keutgen XM . Prevalence and clinical impact of BRAF p.V600E mutation in papillary thyroid carcinoma. Endocr Pathol. (2025) 36:13. doi: 10.1007/s12022-025-09859-y. PMID: 40237893 PMC12003545

[32] Henke LE Perkins SM Pfeifer JD Ma C Chen Y DeWees T . BRAF V600E mutational status in pediatric thyroid cancer. Pediatr Blood Cancer. (2014) 61:1168–72. doi: 10.1002/pbc.24935. PMID: 24677749

[33] Bulanova Pekova B Sykorova V Mastnikova K Vaclavikova E Moravcova J Vlcek P . RET fusion genes in pediatric and adult thyroid carcinomas: Cohort characteristics and prognosis. Endocrine-Related Cancer. (2023) 30:e230117. doi: 10.1530/erc-23-0117. PMID: 37882481 PMC10620462

[34] Lee YA Lee H Im SW Song YS Oh DY Kang HJ . NTRK and RET fusion-directed therapy in pediatric thyroid cancer yields a tumor response and radioiodine uptake. J Clin Invest. (2021) 131:e144847. doi: 10.1530/ey.19.3.13. PMID: 34237031 PMC8439610

[35] Kakudo K Bychkov A Hirokawa M Jung CK Lai C-R Liu Z . Addressing the impact of international variation in thyroid cytology: Which reporting system is best for patients? In: Kakudo K Liu Z Jung CK Hirokawa M Bychkov A Lai C-R , editors.Thyroid FNA cytology: differential diagnoses and pitfalls. Springer Nature Singapore, Singapore (2023). p. 3–6.

[36] Januś D Kujdowicz M Kiszka-Wiłkojć A Kaleta K Taczanowska-Niemczuk A Radliński J . Ultrasound and histopathological assessment of benign, borderline, and Malignant thyroid tumors in pediatric patients: An illustrative review and literature overview. Front Endocrinol. (2024) 15:1481804. doi: 10.3389/fendo.2024.1481804 PMC11821508 39950167

[37] Slack JC Hollowell M Barletta JA . Thyroid nodules and follicular cell-derived thyroid carcinomas in children. Endocr Pathol. (2023) 34:165–75. doi: 10.1007/s12022-023-09764-2. PMID: 37160531

